# Developing onshore wind farms in Aotearoa New Zealand: carbon and energy footprints

**DOI:** 10.1080/03036758.2024.2344785

**Published:** 2024-05-14

**Authors:** Isabella Pimentel Pincelli, Jim Hinkley, Alan Brent

**Affiliations:** a Sustainable Energy Systems, Wellington Faculty of Engineering, Te Herenga Waka Victoria University of Wellington, Wellington, Aotearoa New Zealand; b Department of Industrial Engineering and the Centre for Renewable and Sustainable Energy Studies, Stellenbosch University, Stellenbosch, South Africa

**Keywords:** Wind energy, permanent magnet, blade recycling, greenhouse gas emissions, energy return on investment, life cycle assessment

## Abstract

In recognition of deeper insights into the implications of wind farm deployments, this paper addresses the need for an updated Life Cycle Assessment (LCA) for onshore wind generation systems, using 4.3 MW wind turbines and direct drive permanent magnet synchronous generators. The environmental and energy performances were estimated through an LCA for an onshore wind plant under construction in Aotearoa New Zealand with a total nameplate capacity of 176 MW. This study used real construction data showing literature data overestimates civil works and underestimates transportation contributions in the wind farm footprint. Further, different end-of-life management alternatives for turbine blades are analysed: landfill, mechanical recycling, and chemical recycling. The results indicate a carbon footprint of 10.8–9.7 gCO_2eq_/kWh, a greenhouse gas payback time of 1.5–1.7 years for avoided combined cycle gas turbines, and an energy payback time of 0.4–0.5 years, in which the chemical recycling of the blades is the lower emission solution overall. The outcomes underscore the environmental efficiency of onshore wind farms and their important role in the energy transition. Notably, the manufacturing of wind turbines is the primary contributor to the carbon and energy footprints, highlighting a critical area for targeted environmental mitigation strategies.

## Introduction

Energy systems are going through profound changes, as significant efforts are dedicated to reducing reliance on fossil fuels, increasing energy efficiency, and rapid deployment of renewable energy technologies such as solar and wind generation. Aotearoa New Zealand is committed to the energy transition, with a target of 100% renewable electricity generation by 2030 (Minister of Climate Change 2022), and net zero greenhouse gas emissions by 2050, except for biogenic methane (New Zealand Government 2019). Wind energy is expected to be an essential technology for the expansion of electrification and deployment of renewables in Aotearoa New Zealand. Indeed, all future energy scenarios modelled by different organisations foresee a steep increase in the installed capacity of wind energy generation (Pincelli et al. 2024).

In Aotearoa New Zealand, the deployment of onshore wind plants started in 1993 (Ministry of Business Innovation & Employment 2022), but the wind industry deployment accelerated from 2004 with the adoption of wind turbines with nominal capacities larger than 1 MW (Zhang et al. 2023). By 2023 the onshore wind installed capacity reached 1 GW. The capacity factor of wind generation in the country is very high averaging 41%, almost twice the global average (Zhang et al. 2023), making the country very suitable for wind generation. Indeed, there are several ongoing projects in different stages of execution for new onshore wind plants in Aotearoa New Zealand that could add 2.2 GW to the installed capacity (New Zealand Wind Energy Association n.d.). By 2050, it is estimated the onshore wind installed capacity could reach nearly 6 GW (Pincelli et al. 2024).

The energy transition is further accelerated by the continued decreasing costs of wind generation and other renewable technologies (He et al. 2020). The increase in rotor sizes is a crucial contributor, as larger rotors harness more energy while saving expenses during the operational and management phase and balance of the system (Johnson et al. 2019). Wind turbine technology designs are characterised as either gearbox or direct drive; in the latter, the generator is directly connected to the hub without a gearbox (Das and Nandi 2022). The two technical designs differ significantly in mass and material composition (Carrara et al. 2020). The permanent magnet synchronous generator is the most common direct drive technology (PMS-DD), as it requires low maintenance and has high efficiency. Moreover, the overall weight of wind PMS-DD turbines is lighter, as they do not require gearboxes (Carrara et al. 2020). However, they are more expensive (Moghadam and Nejad 2020). For onshore applications, PMS-DD turbines have been securing a larger market presence, yet their adoption is still not widespread, and the traditional gearbox configuration prevails (Carrara et al. 2020).

Wind energy generates minimal environmental impacts during the operational phase (Das and Nandi 2022). However, wind turbines and other components of an onshore wind plant have embedded environmental footprints associated with their production, as well as plant construction and decommission. Life Cycle Assessment (LCA), a widely used environmental impact assessment method, offers a comprehensive approach to estimate the environmental impacts of products or services throughout their entire life cycle. The carbon footprint and energy requirements of the life cycles of onshore wind plants have been substantially studied within the scientific community. Manufacturing wind turbines has been reported as the major contributor to greenhouse gas (GHG) emissions. However, the results in the literature for the carbon footprint of onshore wind plants vary from 3.3 (Tahtah et al. 2022) to 70 (Nassar et al. 2024) gCO_2eq_/kWh, influenced by different factors such as technology employed, power plant capacity, turbine nominal capacity, site location, and temporal analysis resolution. These considerable variations in reported GHG emissions of onshore wind systems contribute to a lack of consensus on the environmental burdens and benefits of wind systems. It has been previously noticed that, in general, the median values of GHG emissions decrease with the rise in the nominal capacity of wind turbines (Mendecka and Lombardi 2019). However, most LCA studies for onshore wind plants consider wind turbines with a low nominal capacity of up to 2.5 MW, and fewer studies have analysed larger wind turbines (Mendecka and Lombardi 2019).

As the deployment of wind energy rises, the amount of generated turbine waste increases. Thus, ensuring sustainable end-of-life management is crucial. While recycling metals and rare earth elements is feasible, blades, on the other hand, are made of materials that are difficult to recycle, often leading to their disposal in landfills or incineration (Nagle et al. 2020). Technologies for recycling blades have been developed (Yang et al. 2023), but are not feasible yet because of their level of maturity, high costs, or lack of a market for secondary materials (Jensen 2019; Yang et al. 2023). Therefore, assessing the environmental performance of end-of-life management strategies for turbine blades has become relevant, with recent advances in the life cycle benefits of recycling blades (Diez-Cañamero and Mendonza 2023; Sproul et al. 2023; Yang et al. 2023). However, most LCA studies for onshore wind farms have overlooked mechanical and chemical recycling solutions for the blades, considering only a linear production system in which they are disposed of (Das and Nandi 2022; Elmariami et al. 2023; Nassar et al. 2024).

### Objectives of the study

This paper provides a comprehensive LCA of an onshore wind farm under development in Aotearoa New Zealand, and more specifically contributes to updating the environmental performance of onshore wind systems by considering the PMS-DD technology and a nominal capacity of 4.3 MW for the individual wind turbines, as most the literature focuses only on small turbines. Another significant contribution of the study is the utilisation of original real data for the construction phase, including transportation activities. The study enhances the literature by exploring different end-of-life management strategies for blades. Given the predominant linear production system for blades, with recycling efforts only recently emerging, it becomes crucial to assess their environmental performance thoroughly.

## Life Cycle Assessment of onshore wind farms: an overview

LCA studies for wind energy are common, as the technology is mature (Oğuz and Şentürk 2019). Nevertheless, the industry has developed towards larger wind turbines and different designs for generators, and thus, updating the LCA data is crucial and especially so for the Aotearoa New Zealand context. Table 1 summarises the recent and relevant LCA studies conducted for onshore wind farms. The review focused on studies that analysed a complete wind farm system (see Support Information 1 for the literature search).

**Table 1.** Review of relevant and recent LCA studies for onshore wind farms.

Study	Comment	Turbine technology	Turbine capacity	Farm size	Foundation technology	System boundary	Construction phase datasource	EoL for blades	Overall GHG emissions
Nassar et al. (2024)	Considers 10 different turbines, but the technologies are not disclosed	Gearbox	0.85–3.5 MW	100 MW	Made of concrete	Manufacture of components, transport, construction, operation, EoL	Based on the literature	Landfill	32–70 gCO_2eq_/ kWh
Cassoret et al. (2023)	Scenario for onshore wind uptake in France	Gearbox -Ecoinvent database	2 MW	n/a	not disclosed	Manufacture of components, transport, operation, EoL	Based on the literature	Not disclosed	Expressed as percentages
Daaboul et al. (2023)	Characteristics of these turbines are as reported in the Ecoinvent library and are based on Vestas V80 data from 2004.	Gearbox -Vestas V80	2 MW	50 MW	not disclosed	Manufacture of components, diesel for construction & maintenance; electricity for excavation and installation.	Based on the literature	Not disclosed	12 gCO_2eq_/ kWh
Elmariami et al. (2023)		Gearbox -Gamesa G114	2 MW	20 MW	Made of concrete	Manufacture of components, transport, construction, operation, EoL	Not disclosed	Landfill	8.8 gCO_2eq_/ kWh
Tahtah et al. (2023)	Scenarios for using different turbine sizes. Base model similar to Tahtah et al. (2022)	Gearbox -Gamesa G.52-850 kW	Scenarios using: 0.85, 0.8, 1.5, 2, 3 MW turbines	10.2 MW	Made of steel and concrete	Transport, foundation works, assembly, laying and installation of cables	Not disclosed	Incineration	10–16 gCO_2eq_/ kWh
Das and Nandi (2022)		Gearbox and Direct drive design technologies	1.65 MW	56.1 MW	not disclosed	Manufacture of components, construction, maintenance, EoL	Based on the literature	Landfill	Expressed as percentages
Ozsahin et al. (2022)	Analyse scenarios for different metal recycling rates	Gearbox -Nordex	2.5 MW	47.5 MW	Made of steel and concrete	Manufacture of components, construction, O&M, EoL	Original data, however, it is aggregated with manufacturing	Landfill	5.24–7.56 gCO_2eq_/ kWh
Prabhu and Mukhopadhyay (2022)	Scenario for 60 GW of onshore wind installed in India	Vestas V82	1.65 MW	60 GW (installed capacity in India)	Not disclosed	Manufacture of components, installation, O&M, EoL	Not disclosed	Landfill	Presented as total emission
Tahtah et al. (2022)	Simplified LCA	Gearbox -Siemens Gamesa G.52	0.85 MW	12 turbines	Made of steel and concrete	Manufacture of components, transport, installation, O&M, EoL	Not disclosed	Not disclosed	3.3 gCO_2eq_/ kWh
Verma et al. (2022)		Gearbox -Vestas V-82	1.65 MW	10 turbines	Made of steel and concrete	Manufacture of components, transport, installation, O&M, EoL	Not disclosed	Landfill	11.3 gCO_2eq_/ kWh
Li et al. (2021)	Considered impacts of wind farm infrastructures and work in detail	Not disclosed	2 MW	40 MW	Reinforced concrete	Manufacture of components, transport, installation, O&M, EoL	Original data	Landfill	16.4–28.2 gCO_2eq_/ kWh
Şentürk et al. (2021)	Compared solar PV, onshore, and offshore wind. Onshore wind system relates to Oğuz & Şentürk et al. (2021)	Direct drive -Enercon E-40	0.6 MW	10.2 MW	Made of steel and concrete	Manufacture of components, transportation, O&M	Not disclosed	Not included	10.64 gCO_2eq_/ kWh
Thopil (2021)	Used Ecoinvent dataset	Not disclosed	2.3 MW	138 MW	Not disclosed	Manufacture of components, transport, installation, O&M, EoL	Based on the literature	Not disclosed	6.6 gCO_2eq_/ kWh
Vélez-Henao and Vivanco (2021)		No drivetrain info. -Nordex N60	1.3 MW	19.5 MW (15 turbines)	not disclosed	Manufacture of components, installation, O&M, EoL	Based on the literature	Landfill	12.93 gCO_2eq_/ kWh
Basosi et al. (2020)	Compared wind, solar, and geothermal generation	-Repower MM92	2 MW	18 MW	Not disclosed	Manufacture of components, installation, O&M, EoL	Original data	Recycling	Presented as mid points.
Alsaleh and Sattler (2019)	Installation phase is comprehensive.	Gearbox -Siemens Gamesa G83 and G87	2 MW	400 MW	Made of steel and concrete	manufacturing; transportation; installation; O&M; EoL	Original data	Recycling	18 gCO_2eq_/ kWh
Gomaa et al. (2019)		-Vestas V112	3 MW	38 turbines	Made of steel and concrete	Manufacture of components, transport installation, O&M, EoL	Original data	Landfill	9.11 gCO_2eq_/ kWh
Oğuz and Şentürk (2019)	Compared onshore wind to solar PV.	Gearbox -Enercon E-40	0.6 MW	10.2 MW	Made of steel and concrete	Manufacture of components, construction, O&M, EoL	Based on the literature	Landfill	10.58 gCO_2eq_/ kWh
Gaete-Morales et al. (2018)	Analyse the electricity system in Chile	No drivetrain info. -Vestas	2 MW	16 farms, with a total capacity of 831 MW	not disclosed	Wind turbine, power plant construction, O&M, dismantling	Not disclosed	Landfill	8 gCO_2eq_/ kWh
Ozoemena et al. (2018)	Analyse scenarios for advancements in rotor, tower concepts, and drivetrain (including single-stage gearbox).	Direct drive -Enercon E-66 (baseline turbine)	1.5 MW	114 MW	Made of steel, concrete and PVC	components manufacture, site construction, O&M, dismantling	Estimations	Disposal facility	11.8–16.6 gCO_2eq_/ kWh
Xu et al. (2018)		No drivetrain info. -Goldwind GW77 and S50	1.5 and 0.75 MW	49.5 MW	Made of steel-reinforced concrete and concrete foundations	components manufacture, installation, O&M, EoL	Original data	Landfill & Incineration	8.65 gCO_2eq_/ kWh
Atilgan and Azapagic (2016b)	Electricity system in Turkey. Similar to Atilgan and Azapagic (2016a)	No drivetrain info. -Vestas V80	1.94 MW	39 farms, with a total capacity of 1320 MW	not disclosed	Wind turbine construction, operation, decomission	Not disclosed	Landfill	7.3 gCO_2eq_/ kWh
Atilgan and Azapagic (2016a)	Electricity system in Turkey.	No drivetrain info. -Vestas V80	1.94 MW	39 farms, with a total capacity of 1320 MW	not disclosed	Wind turbine construction, operation, decomission	Not disclosed	Landfill	7.3 gCO_2eq_/ kWh
Bonou et al. (2016)		Gearbox and direct drive -Siemens	2.3 and 3.2 MW	20 turbines	Steel reinforced concrete.	Manufacture of components, transport installation, O&M, EoL	Original data	Incineration	5 and 6 gCO_2eq_/ kWh
Portugal-Pereira et al. (2016)	Simplified LCA. Incorporate LCA results into power generation optimisation	not disclosed	not disclosed	4 MW	not disclosed	Cradle to gate	Not disclosed	Not included	4.37 gCO_2eq_/ kWh
Al-Behadili and El-Osta (2015)		Direct drive -Mtorris TWT 1.65/82	1.65 MW	60 MW	not disclosed	Manufacture of components, installation, O&M, EoL	Based on the literature	Not disclosed	10.4 gCO_2eq_/ kWh
Garrett and Rønde (2013)	Analyse electricity generation depending on wind speeds	Gearbox -Vestas Grid Streamer™	2 MW	50 MW	not disclosed	Cradle to grave	Original data	Disposal	7.2–9.7 gCO_2eq_/ kWh
Oebels and Pacca (2013)		not disclosed	1.5 MW	14 turbines	not disclosed	Material processing, components manufacture, site construction, O&M, EoL	Original data	Not disclosed	7.1 gCO_2eq_ /kWh
Rajaei and Tinjum (2013)		Gearbox -Vestas V90	1.8 MW	162 MW	Made of steel and concrete	cradle-to-gate	Based on the literature	Not included	16.9 gCO_2eq_/ kWh
Rashedi et al. (2013)	Analyse horizontal and vertical turbines.	Gearbox -Repower horizontal turbine,	5 MW	50 MW	Concrete and steel foundation for horizontal turbine, vertical turbine has simpler foundation made only with steel	Material processing, components manufacture, site construction, O&M, EoL	Based on the literature	Landfill	Expressed as points

Most studies considered a system boundary from cradle-to-grave, including material extraction and processing, component manufacture, transportation, installation and construction, operation and maintenance, dismantling, and end-of-life management. However, few studies comprehensively included the impacts of wind farm infrastructure construction and installation, such as civil and electrical works and project management (Alsaleh and Sattler 2019; Li et al. 2021). Other studies rely on the Ecoinvent database for modelling the wind farm components and phases. However, that might lead to a potential overestimation of certain impacts because of the rapid evolution of the technologies (Cassoret et al. 2023).

The literature review shows that despite industry advancements in increasing wind turbine sizes, most LCA studies have considered turbines up to 2 MW of nominal generation capacity (see Table 1). The environmental performance of different wind turbine technology designs has been previously analysed (Schreiber et al. 2019). However, apart from the reported research of Das and Nandi (2022) and Şentürk et al. (2021), most reviewed LCA studies for onshore wind farms only considered wind turbines with gearboxes or overlooked the design technology.

Specific within the Aotearoa New Zealand context, Rule et al. (2009) estimated the life cycle GHG emissions and energy demand for wind energy systems. More recently, a simplified LCA model has been proposed to generate energy and carbon indicators, which is based on correlations of the dimensions and weights of the components of turbines rather than a comprehensive process-based LCA (Walmsley et al. 2017).

The Siemens-Gamesa and Vestas wind turbine manufacturers offer LCA or Environmental Product Declaration (EPD) analyses for their wind turbines, encompassing various turbine sizes, including larger ones. However, for onshore applications, their considerations are limited to the gearbox technology design only.

Another limitation observed in prior LCA studies conducted for onshore wind farms is the exclusion of alternative end-of-life management strategies for wind turbine blades beyond landfilling and incineration (see Table 1). Basosi et al. (2020) and Alsaleh and Sattler (2019) assumed a recycling rate for blades, yet notably omitted substantial information regarding the recycling process technologies employed, the underlying assumptions and data utilised, nor did they elaborate on the results and discussion about blade recycling. Thus, those previous studies lack crucial insights into the frameworks and implications of recycling blades.

Recent efforts have been made to understand the environmental performance of alternatives for the end-of-life management of blades through specialised LCA studies focusing specifically on this aspect (Diez-Cañamero and Mendonza 2023; Sproul et al. 2023; Yang et al. 2023). Recycling technologies for post-consumer blades, such as mechanical and chemical recycling, remain infeasible due to varying levels of maturity, cost competitiveness, and market availability (Yang et al. 2023). Nevertheless, it is anticipated that these technologies will advance over time. Therefore, it is crucial to incorporate them into LCA studies for onshore wind farms to accurately evaluate their potential environmental benefits and inform decisions.

Summarising, the conducted literature review reveals gaps that limit a comprehensive understanding of the environmental impacts of wind farms. First, studies tend to overlook the implications of larger turbines, which are becoming increasingly prevalent in the wind energy sector. Second, the choice of turbine technology is also commonly disregarded, and greater attention needs to be paid to permanent magnet direct drive generators. Another significant oversight is the lack of investigation into blade recycling routes, despite the growing importance of sustainable end-of-life management within the wind energy sector. This study addresses these gaps by contributing to the development of a more comprehensive LCA for wind farms.

### Review of results for life cycle greenhouse emissions

**Figure 1.**
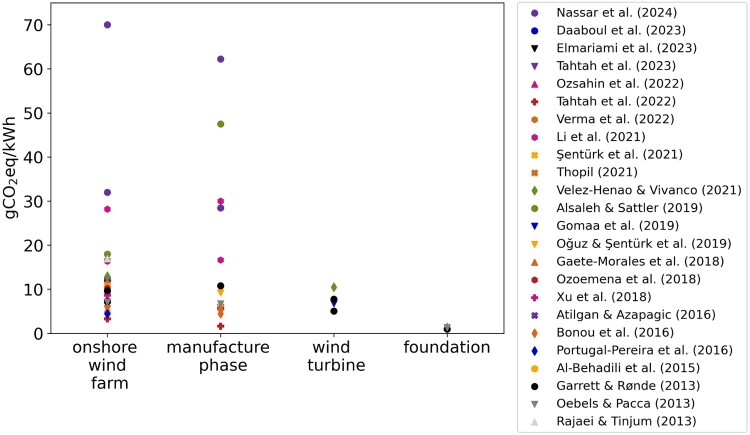
Results from the literature for overall life cycle GHG emissions of onshore wind farms and the contributors to the emissions. Some values were approximated.

The reviewed lifecycle GHG emissions for onshore wind farms, as mentioned before, vary from 3.3 (Tahtah et al. 2022) to 70 (Nassar et al. 2024) gCO_2eq_/kWh (see Figure 1 and Table 1), underscoring the diverse carbon footprints across different studies in the literature. An even higher variability for life cycle GHG emissions, for onshore micro-sized applications, has been reported (Mendecka and Lombardi 2019). This variability can be attributed to a range of factors, including different farm sizes, turbine technology and nominal capacity, capacity factors, and the modelling methodologies employed. Nevertheless, it has been indicated that the median value for GHG emissions for onshore wind farms stood at 9.7 gCO_2eq_/kWh (Mendecka and Lombardi 2019).

The manufacturing phase has been reported as the primary contributor to GHG emissions (Figure 1). Materials extraction and processing, and the production of wind turbines encompass approximately 90% of the life cycle GHG emissions of onshore wind farms (Kadiyala et al. 2017).

## Method

**Figure 2.**
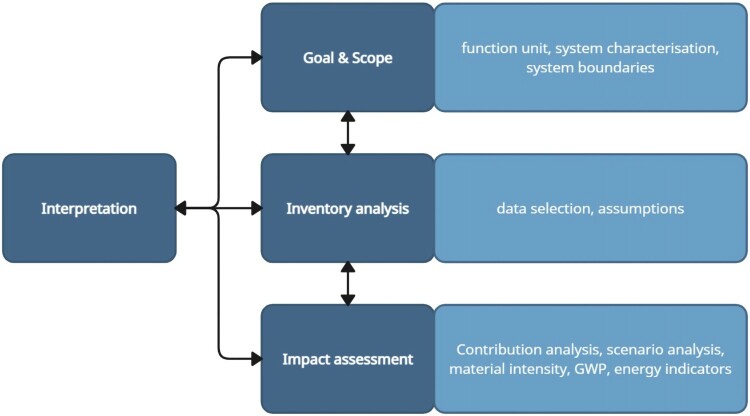
Life cycle assessment (LCA) framework, based on ISO 14040 (2006).

The LCA approach was used to assess the potential GHG emissions and cumulative energy demand (CED) of a 176 MW onshore wind farm, employing 4.3 MW turbines, under construction in Aotearoa New Zealand. The LCA is a standardised method defined by ISO 14040 and ISO 14044 to evaluate potential environmental impacts caused by a product, process, or service over its entire life cycle. The LCA framework consists of defining the goal and scope of the study, analysing the inventory data, assessing environmental impacts, and interpreting the results (see Figure 2). The LCA was modelled using the Activity-Browser software package (Steubing et al. 2020).

The functional unit is 1 kWh of electricity generated (assuming a 30-year operational life).

### System description

**Figure 3.**
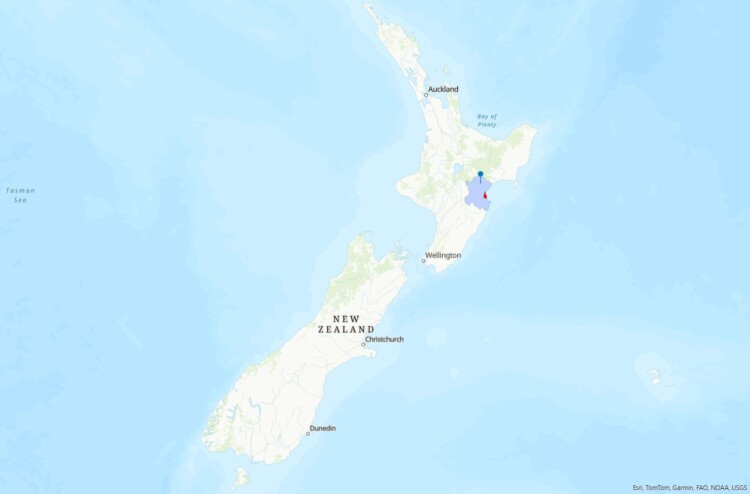
Location of the developed wind farm pinpointed, in Hastings District, in light blue, 35 km northwest of Napier City, in red.

The analysed onshore wind farm is the Harapaki Wind Farm, developed by Meridian Energy Ltd. The farm is under construction in the Hastings District of Hawke’s Bay, approximately 35 km northwest of Napier Port (Meridian Energy n.d.) (see Figure 3).

The wind farm comprises 41 turbines, with 176 MW installed capacity. The wind farm is expected to generate 452 GWh per year and has a thirty-year operating life. The plant layout consists of 6 strings of 5–8 turbines (Meridian Energy n.d.). The key parameters of the onshore wind system are presented in Table 2. The specifications of the components of the wind farm and the technical parameters of the wind turbines are presented in Tables 3 and 4, respectively. Information was retrieved from consulting Meridian Energy Ltd., as well as their website and publicly available documents.

**Table 2.** Key parameters of the onshore wind plant.

Parameter	Description	
System capacity	176	MW
System lifetime	30	years
Estimated capacity factor	35	%
Mean annual electricity generation	542	GWh/year
Electricity generation over lifetime	16,260	GWh

**Table 3.** Specifications of the components for the onshore wind plant.

Specification		Value
Wind turbine	Siemens Gamesa SWT-DD-120	41 turbines
Foundation	Piles, concrete and steel, reduced concrete	
Transformers		2 transformers
Cabling		250 km length
O&M building		2 switch-rooms and a building with warehouse and offices
Road access		24 km length

**Table 4.** Technical specification for the wind turbines.

Specifications		
Nominal capacity	4.3 MW	
Generator technology	Permanent magnet synchronous	
Drive train	Direct drive	
Number of blades	3	
Tower technology	Tubular steel tower	
Rotor diameter	120	m
Blade length	60	m
Hub height	85	m

**Figure 4.**
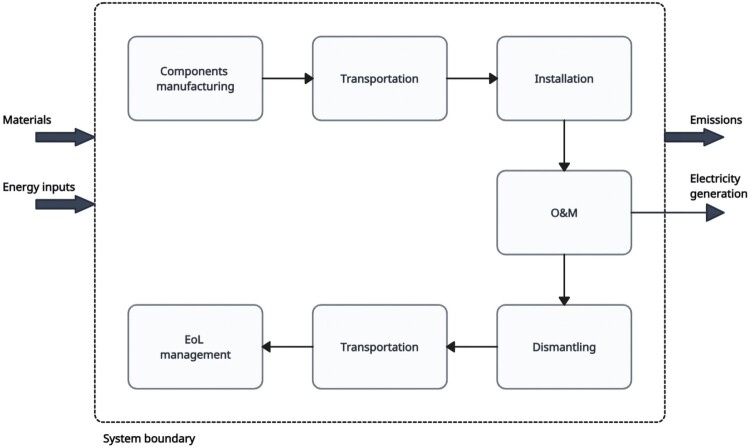
LCA system boundary adopted for the onshore wind farm study.

The boundary of the study is from cradle-to-grave (see Figure 4). The wind towers, nacelles, and rotors are assumed to be manufactured in China, the transformers, in South Korea, and the cables, in China. The imported components enter Aotearoa New Zealand at Napier Port, close to the wind farm site.

### Inventory

The wind turbine technology is direct drive, with a permanent magnet synchronous generator (PMS-DD). Data for the material composition were collected from Carrara et al. (2020), which presents specific data for this wind turbine technology. Table 5 presents the inventory for the wind turbine, in which the tower is mostly made of large tubular steel sections, the nacelle contains the electrical and mechanical components, including the permanent magnet generator manufactured utilising rare earth elements, and the blades, mainly made from glass and carbon fibres infused with epoxy resin (Carrara et al. 2020).

**Table 5.** Inventory for PMSG-DD wind turbine manufacturing.

Specification	Value		Source
*Material Intensity*			
Steel	121,164	kg/MW	Carrara et al. (2020)
Copper	3,000	kg/MW	Carrara et al. (2020)
Aluminium	500	kg/MW	Carrara et al. (2020)
Iron	50,275	kg/MW	Carrara et al. (2020)
Neodymium	774	kg/MW	Carrara et al. (2020)
Dysprosium	73	kg/MW	Carrara et al. (2020)
Boron	26	kg/MW	Carrara et al. (2020)
Praseodymium	151	kg/MW	Carrara et al. (2020)
Terbium	30	kg/MW	Carrara et al. (2020)
Zinc	23,650	kg/MW	Carrara et al. (2020)
Glass fibre in the nacelle	3,083	kg/nacelle	Ecoinvent v.3.9.1
Glass fibre in the set of blades	27,567	kg/set of blades	Carrara et al. (2020) & Ecoinvent v.3.9.1
Carbon fibre	1,782	kg/MW	Carrara et al. (2020)
Epoxy resin	4,600	kg/MW	Carrara et al. (2020) & Li et al. (2022)
*Energy Intensity*			
Electricity	0.5	kWh/kg	Brussa et al. (2023) & Ecoinvent v.3.9.1

Inventory data for the material composition of the cables were taken from product specifications. The data for the transformer was approximated with the transformer used in the study of Vélez-Henao and Vivanco (2021). Support information 2 presents the data utilised for manufacturing cables and transformers.

The foundation is made of concrete and steel. The concrete is produced on-site, assuming that steel and cement are sourced mainly in the Hawkes Bay region. The wind farm project reduced the concrete and steel requirement for the foundations (Batters 2023). The data for the foundation were collected from the farm developers and aggregated within civil works during the construction phase.

The site construction work constitutes soil removal for the tower installation, mounting mobile cranes, concrete foundations, access road construction, laying and installation of cables, and usage of energy and electricity for other purposes. Detailed data were obtained directly from the wind farm developer for civil works, electrical works, project management, and turbine works. The data specified the material demand, fuel consumption in equipment, electricity usage, and waste management.

For the operational and maintenance (O&M) phase, the following activities were considered: change of lubricants and motor oils, and turbine inspections. Turbine inspections were assumed to occur twice a year per turbine in a passenger vehicle (Vélez-Henao and Vivanco 2021). It is assumed that 33% of the blades, and 15% of the generator, nacelle, and hub system are replaced over the lifetime (Vélez-Henao and Vivanco 2021).

Wind turbines are decommissioned when the onshore wind farm reaches the end-of-life. In the decommissioning phase, foundations and access roads are assumed to remain on site. It was assumed that decommissioning the farm requires the same amount of electricity and mounting mobile crane usage as for constructing it.

The components are dismantled, the recyclable materials are recycled, and the non-recyclable waste is disposed of in landfills. Recycling some of the turbine components and materials is relatively simple because the recycling processes for those materials are already established. Metals and bulk materials, such as steel, copper, and aluminium, are assumed to be recycled with a recycling rate of 90%. Recycling rare earth elements has been minimal across different industry sectors (Jensen 2019). However, recycling rare earth elements from permanent magnets in wind turbine generators is more feasible because of their large size, accessibility, and disassembly procedures (Jensen 2019). A recycling rate of 81% is assumed for neodymium, dysprosium, and boron, as it is economically viable to recycle large amounts of permanent magnets (Reimer et al. 2018). The composite materials used for making the blades are the most difficult to recycle.

Although recycling of the blades is not feasible yet because of high costs and the lack of a market for secondary materials (Jensen 2019), scenarios for mechanical and chemical recycling were produced as those solutions are emerging. Mechanical recycling consists of reducing the blade size to generate powder and fine fibre fractions (Sorte et al. 2023; Yang et al. 2023). While recycled fibre can be used to replace glass fibre, the powder can substitute calcium carbonate filler (Yang et al. 2023). Chemical recycling by the solvolysis process decomposes the polymer from the composite by utilising a liquid solvent. The fibres and resins from the blades are recovered in the chemical recycling process. Table 6 presents the parameters used for each end-of-life management solution of the blades.

**Table 6.** Parameters for the end-of-life management solutions of the blades.

Scenario	Parameter	Value		Source
*Landfill*	*Input*			
	Energy for material crushing	0.22	MJ/kg	Diez-Cañamero and Mendonza (2023)
*Mechanical recycling*	*Input*			
	Energy for material crushing	0.22	MJ/kg	Diez-Cañamero and Mendonza (2023)
	Energy for material shredding	0.04	MJ/kg	Diez-Cañamero and Mendonza (2023)
	Energy for the recycling process, griding	0.27	MJ/kg	Diez-Cañamero and Mendonza (2023) & Sproul et al. (2023)
	*Output*			
	Recycled fibre fraction	42	%	Yang et al. (2023)
	Recycled powder fraction	30	%	Yang et al. (2023)
	Coarse fraction	28	%	Yang et al. (2023)
*Chemical recycling*	*Input*			
	Energy for material crushing	0.22	MJ/kg	Diez-Cañamero and Mendonza (2023)
	Energy for material shredding	0.04	MJ/kg	Diez-Cañamero and Mendonza (2023)
	Energy for the recycling process	6.47	MJ/kg	Yang et al. (2023)
	Deionised water	1.35	kg/kg	Yang et al. (2023)
	Acetic acid	0.45	kg/kg	Yang et al. (2023)
	Sodium hydroxide	0.04	kg/kg	Yang et al. (2023)
	*Output*			
	Recycled fibre	0.6	kg	Yang et al. (2023)
	Recycled resin	0.58	kg	Yang et al. (2023)
	Phenol	0.29	kg	Yang et al. (2023)
	Trimethyl benzene	0.08	kg	Yang et al. (2023)
	Aniline	0.03	kg	Yang et al. (2023)

For the background data, the Ecoinvent v.3.9.1 database was utilised. The electricity grid mix and industrial heat specific for the regions where the components are manufactured were used.

### Impact assessment

The climate change impact category was selected to analyse the environmental impacts of the onshore wind farm, for which the indicator global warming potential (GWP100) was used. The GWP measures lifecycle GHG emissions in kg CO_2eq_, using the IPCC (GTP100) method. The cumulative energy demand (CED) was also included as part of the lifecycle energy analyses.

The GHG payback time (GPBT) metric was calculated, which represents the period the system must operate to offset the emissions embedded in its lifecycle, per Equation (1).

(1) $$G\mathrm{PBT}=\;\frac{G\mathrm{HG}\;\mathrm{emission}s_{L\mathrm{CA}}}{A\mathrm{nnual}\;\mathrm{GHG}\;\mathrm{emission}s_{a\mathrm{voided}}}$$

The GPBT was calculated considering avoided emissions from consuming electricity from the grid (Fonseca and Carvalho 2022), as well as from gas turbines, as in the Aotearoa New Zealand context the generated electricity of the onshore wind system potentially displaces electricity dispatched from combined-cycle gas turbine power plants.

The energy performance was measured using the cumulative energy demand (CED), energy payback time (EPBT), and energy return on investment (EROI) metrics. The CED is the total primary energy harvested from nature to supply the onshore wind system; it is the sum of the primary energy demand to produce materials, manufacture and transport the components, and install, operate, and for the end-of-life management of the system. The EPBT is the time period (years) for the onshore wind system to generate the same amount of energy that was used to produce the system, operate it, and manage its end-of-life (Fthenakis 2017), calculated utilising Equation (2).

(2) $$E\mathrm{PBT}=\;\frac{C\mathrm{ED}}{\left(\left(\frac{E_{g\mathrm{eneration}}}{\eta_{G}}\right)-E_{O\&M}\right)}$$

where, E_generation_ is the mean annual electricity generation (MJ_el_/year), EO&M is the annual energy demand for operation and maintenance, and ηG is grid efficiency.

EROI represents how much energy is obtained from a system compared to how much of that energy is required to create and implement the system. It is described by a unitless ratio of the energy returned to society to the energy required to make that energy source (Fthenakis 2017).

## Results and discussion

**Figure 5.**
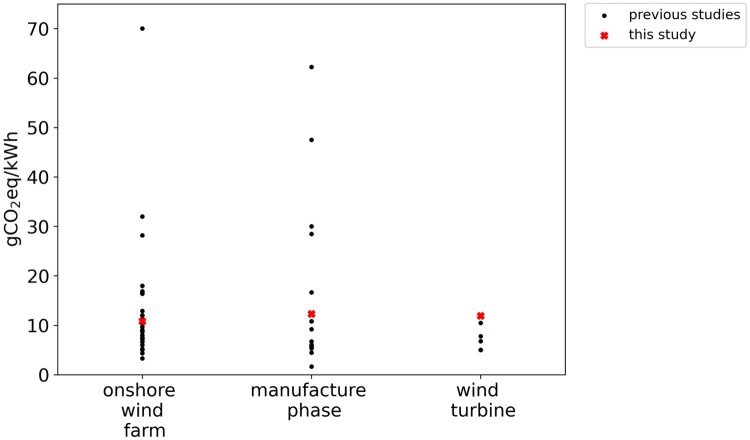
Overall life cycle GHG emissions of the onshore wind farm in Aotearoa New Zealand, compared to previous studies in the literature.

The estimated life cycle GHG emissions for the onshore wind system is 10.8 gCO_2eq_/kWh. While GHG emission footprints for onshore wind systems are very diverse in the literature, this study results sit within the range reported in the literature (see Figure 5).

Comparing lifecycle GHG emissions of onshore wind farms must be approached with some caution, because of different assumptions, methodological choices, modelled technologies, and site specifications. Moreover, the variability for onshore wind farms is significantly high. For instance, the GHG emissions for another wind farm deployed in Aotearoa New Zealand were estimated as 3 gCO_2eq_/kWh (Rule et al. 2009), much lower than this study’s wind farm case. According to the review of Mendecka and Lombardi (2019), the overall median value for onshore wind farms is 9.7 gCO_2eq_/kWh. The life cycle emissions result of this study is, therefore, aligned with the median carbon footprint reported in the literature.

The environmental payback time, in terms of GPBT, for the onshore wind farm is 3.1 years (avoiding the national grid) and 1.7 years (avoiding combined cycle gas turbines). The electricity grid in Aotearoa New Zealand is already of low carbon intensity, and its mean emission factor over the last 5 years is 103 gCO_2eq_/kWh (Ministry for the Environment 2022). Nevertheless, the onshore wind system emissions are 89.5% lower compared to the country’s grid. Deploying onshore wind plants in Aotearoa New Zealand therefore helps to reduce the emissions associated with the energy system as fossil fuels are phased out and electrification expands.

This study has some methodological limitations. First, it focuses only on the energy intensity and GHG emissions throughout the life cycle of the wind farm, even though there are other environmental impacts, such as ozone depletion, human toxicity, acidification, eutrophication, and resource depletion. Social, wildlife, or economic impacts were not considered. In addition, this LCA study estimates potential impacts, not measuring real impacts on the field. The results are limited by available inventory data, although data were selected representing the specific technology (4.3 MW permanent magnet direct drive turbine), and original data were utilised for construction and transportation phases.

### Energy indicators

The findings indicate that the system is highly energy efficient. The onshore wind plant recoups the energy used in its production in 0.5 years, providing a quick net energy benefit. The low EPBT value indicates that the system enhances environmental benefits, as it generates much more energy than was consumed during its production. The EROI result is 66, which shows a high energy gain compared to the energy invested to manufacture the onshore wind plant. The EROI of onshore wind plants in Aotearoa New Zealand has been previously estimated, reaching up to 62.3, depending on the plant (Walmsley et al. 2017). The findings show that onshore wind plants are very efficient providing significant net energy gain and contributing to long-term sustainability.

### Contribution analysis

**Figure 6.**
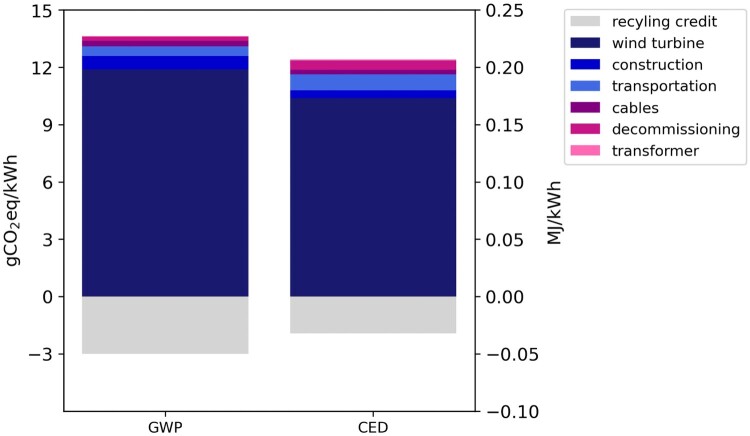
Life cycle GHG emissions and CED for the analysed wind plant in Aotearoa New Zealand.

The largest estimated contributor to GHG emissions and CED is the manufacturing of wind turbines (see Figure 6). This outcome is consistent with the findings in the literature. The GHG emissions are driven by the large mass share of the turbines, along with the energy and carbon-intensive processes involved in producing and processing essential materials to manufacture them.

Within the turbine’s manufacturing process, the usage of steel was identified as the main source responsible for nearly half of the emissions. Steel production is energy-intensive, coming from non-renewable heating sources, such as coal furnaces, and electricity mix. A fifth of the emissions associated with turbine manufacturing stems from the blades, primarily due to the production processes of carbon and glass fibres. Wind farms can enhance their environmental performance by minimising the steel requirement for towers through advancements in design and incorporating recycled secondary steel materials in manufacturing (Vélez-Henao and Vivanco 2021). Additionally, the wind energy sector has been developing new technologies for blade production, including utilising organic materials (Li et al. 2022). Support Information 3 presents a sensitivity analysis for the demand for steel, glass and carbon fibres, as well as for the required electricity for assembly.

Increasing the usage of renewable energy sources in the manufacturing phase can significantly enhance overall emissions reduction for future wind farm developments. Given that most components are manufactured in China, the country’s ongoing energy transition holds the potential to reduce the embodied emissions associated with wind turbines.

The GHG emissions credit for recycling metals in the end-of-life management phase of turbines and substations contributes significantly to the reduction of the overall life cycle emissions of the farm by 28% (−3.0 gCO_2eq_/kWh). Other studies have highlighted that recycling materials at the wind farm end-of-life phase can yield GHG emissions savings ranging from 20 (Bonou et al. 2016) to 40% (Atilgan and Azapagic 2016b). Recycling credits recognise that recycling materials avoid the extraction of raw materials and their associated environmental impacts. Incorporating recycling routes, therefore, increases the environmental performance of the wind farm.

The life cycle phases of a wind farm hold different levels of uncertainty. The manufacturing, installation and transportation phases hold more certainty, the former because of well-established manufacturing processes, and the latter because of the implementation of original data, which is discussed in the following subsection. However, uncertainties arise in the end-of-life phase. Wind farms have long lifetimes, and extensive experience with end-of-life management for wind farm components is still lacking. The effectiveness of recycling routes and their associated environmental impacts might change over time with more experience in managing the end-of-life of wind farms.

### Construction and transportation phases

**Figure 7.**
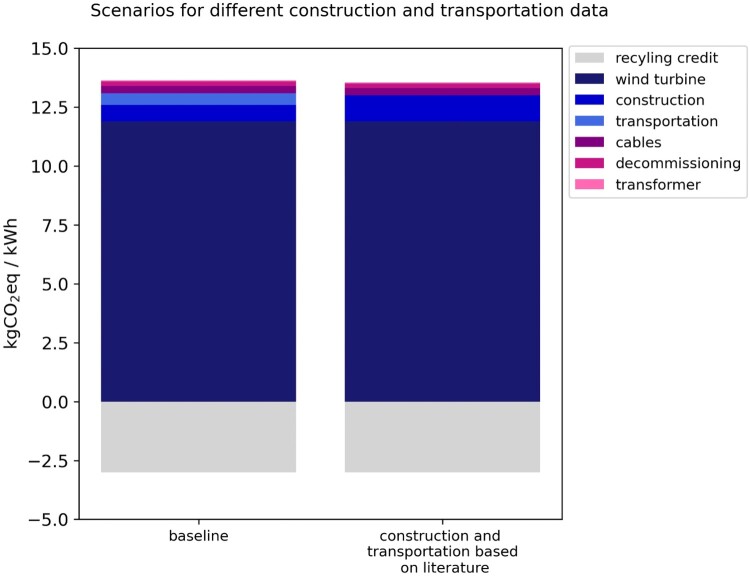
Life cycle GHG emissions for the analysed wind plant in Aotearoa New Zealand, comparing data for construction and transportation phases.

The environmental impacts of the installation and transportation phases are important. Together they accounted for nearly 10% of the overall emissions. However, they are less commonly addressed in the literature. The comparison of the construction and transportation phases utilising different databases, namely, original data and data estimated based on the literature, reveals interesting insights, presented in Figure 7.

When employing original data sourced directly from the wind farm developer, the construction phase exhibits lower emissions, attributed to reduced concrete usage and optimised construction methods. On the other hand, the transportation phase demonstrates higher emissions, reflecting the inclusion of all contractors’ transportation activities. This highlights the significance of comprehensive data collection to assess environmental impacts accurately. The scenario reliant solely on literature values may underestimate transportation and overestimate construction emissions due to the variability in practices across projects.

LCA using original data for the installation and services of wind farms has also indicated that environmental impacts were largely associated with transportation, as in the Bonou et al. (2016) study. Strategies for achieving environmental improvements for the transportation phase include the adoption of electrified vehicles, incorporation of renewable fuels, and optimisation of logistic processes. Optimising logistics can reduce travel and fuel consumption through route planning, load consolidation, and vehicle sharing.

Other LCA studies have reported a higher emission share for the installation phase attributed to the production of cement for the concrete foundation (Gomaa et al. 2019). In this study, during the construction phase, cement, steel, and gravel production are the main contributors to GHG emissions. Strategies to enhance the environmental performance of the installation phase include increasing the utilisation of recycled crushed materials on roads, increasing the fuel efficiency of heavy machinery (Rajaei and Tinjum 2013), and incorporating alternative cementitious materials.

### Blade end-of-life management scenarios

**Figure 8.**
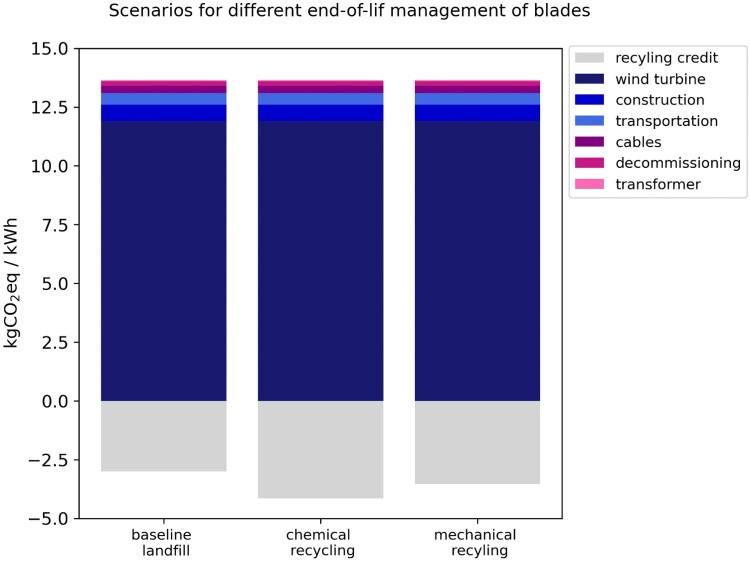
Life cycle GHG emissions for the analysed wind plant in Aotearoa New Zealand, considering different end-of-life management strategy for the blades.

The life cycle assessment findings for end-of-life management strategies of turbine blades reveal recycling post-consumer blades reduces the wind farm’s overall GHG emissions compared to the conventional landﬁll management route (see Figure 8). The LCA findings disclose life cycle emissions considering different end-of-life management solutions for the blade: 10.8 gCO_2eq_/kWh for landfilling (baseline scenario), 10.3 gCO_2eq_/kWh for mechanical recycling, and 9.7 gCO_2eq_/kWh for chemical recycling. Chemical recycling offers a slight advantage in terms of carbon emissions savings over both landfilling and mechanical recycling. However, it is crucial to acknowledge that chemical recycling is still on an experimental scale, whereas mechanical recycling is an existing mature technology (Yang et al. 2023).

Although LCA studies for wind farms overlook the recycling of blades, this study results align with the LCA findings of Diez-Cañamero and Mendonza (2023) specifically for end-of-life management of turbine blades, in which chemical recycling potentially generates higher carbon credits. However, there remains no consensus on the environmental impacts and benefits of chemical recycling, as other studies have yielded higher GHG emissions values due to increased energy consumption and potential sensitivity to thermal energy sources (Sproul et al. 2023). Chemical recycling is currently in a laboratory demonstration phase, suggesting that its GHG emissions may change as the process transitions to larger-scale implementation.

In this study, the mechanical recycling process is inherently more energy-intensive compared to the other end-of-life management solutions. The environmental impact intensity of each end-of-life management route is notably sensitive to changes in the energy mix. Thus, shifting towards renewable electricity generation can significantly reduce the emissions associated with waste recovery methods such as mechanical recycling. Given that mechanical recycling is a more mature technology, it holds promise for effective end-of-life management for the post-consumer blades, moving away from conventional disposal in landfills.

Developing recycling routes aimed at reducing the environmental impacts of post-consumer blades at their end-of-life stage is therefore imperative. Feasible, large-scale recycling routes are required for turbine blades, aligning with the objectives of the electricity transition and the circular economy. As these technologies mature, become cost-competitive, and attain commercial availability, they hold the potential to enhance the environmental performances of wind farms.

Other options for mitigating the impacts at the end-of-life stage of turbine blades can involve exploring secondary uses for the blade, such as pedestrian bridges and transmission towers. However, they are less demanding applications not capable of addressing all the potential post-consumer blades (Yang et al. 2023). Other end-of-life management options, such as incineration, pyrolysis, and co-generation in cement kilns, were not assessed in this study and could be subject to further investigation in subsequent research endeavours.

## Conclusion and prospects

As Aotearoa New Zealand undergoes a profound shift towards low-carbon energy solutions, with the expansion of electrification and deployment of wind systems, it becomes increasingly important to adapt and update LCA for these systems to comprehensively evaluate their life cycle energy demand and GHG emissions. This study has shown that despite energy investments and GHG emissions in the production phase, the onshore wind plant offsets its emissions over its lifespan, making it a suitable option for the energy transition in Aotearoa New Zealand, and elsewhere. This underscores that onshore wind plants are aligned with the principles of sustainable development. Nevertheless, it remains crucial to continue implementing improvements aimed at limiting negative environmental impacts while maximising positive contributions throughout the supply chain of onshore wind plants.

Recycling post-consumer turbine blades is emerging as an alternative to address the challenge of blade waste, which is currently predominantly disposed of in landfills. Both mechanical and chemical recycling methods show potential environmental benefits, reducing the overall wind farm GHG emissions from 10.8 (landfill) to 10.3 and 9.7 gCO_2eq_, respectively. However, it is crucial to note that these recycling technologies are not yet commercially feasible, and mechanical recycling presents the most mature option. As recycling processes continue to evolve, their potential environmental impacts and benefits are subject to change, particularly as they become more efficient and utilise renewable electricity sources in their operations. Therefore, ongoing research and development efforts are essential to analysing the potential benefits of recycling turbine blades and integrating them into LCA for onshore wind farms.

The prospects for further LCA studies for wind farms rely on accounting for ongoing changes in the energy supply, end-of-life management, and technology advances. As manufacturing countries undergo an energy transition and increasingly rely on renewable sources, understanding the implications of these shifts on the environmental footprints of wind turbines is crucial. As the wind energy sector gains more expertise in end-of-life management, adjusting LCA databases to accurately reflect these developments is required. Finally, because of the rapid advancements of technologies, regular updates to LCA studies are necessary to ensure they remain reflective of current practices and accurately inform decision-making processes.

## Supplementary Material

Supplemental Material
