# Supplementary material for: Developing onshore wind farms in Aotearoa New Zealand: carbon and energy footprints
Source: J R Soc N Z. 2024 May 14;55(4):1005–27. doi: 10.1080/03036758.2024.2344785 (PMC12054583; doi:10.1080/03036758.2024.2344785)

**Support Information 1**

Figure S1: Literature search.


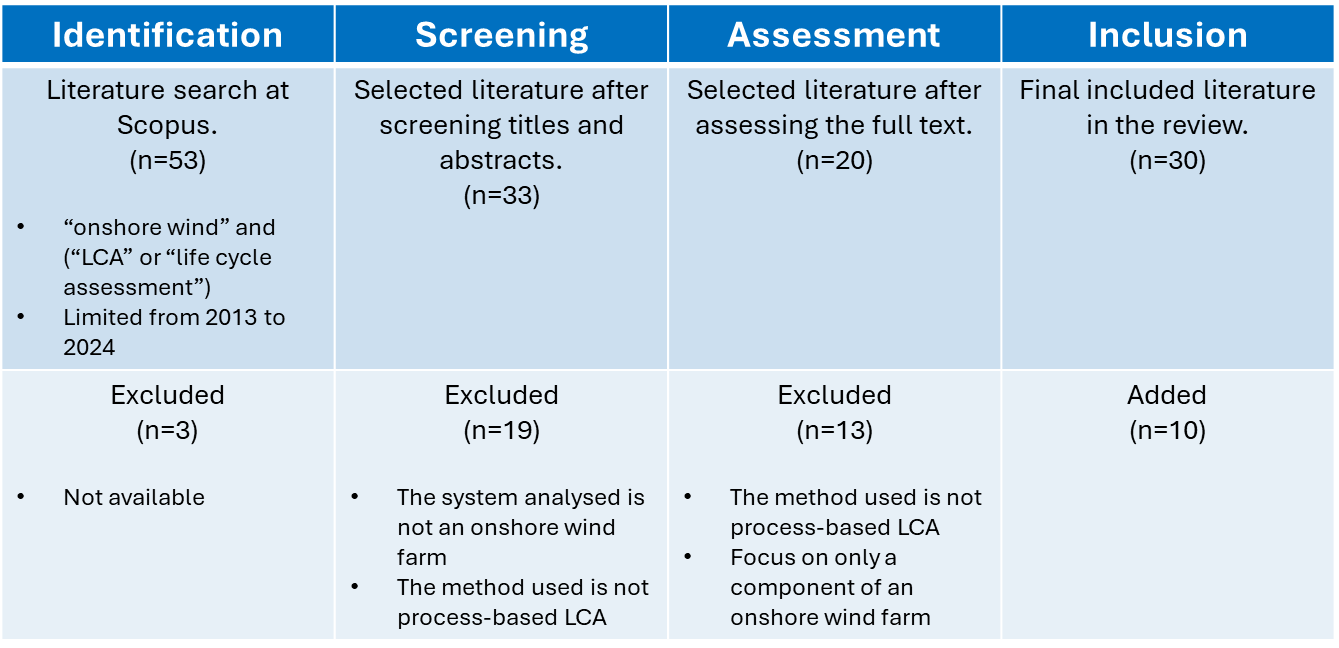


**Support Information 2**

Table S2.1. Inventory for the low voltage cable.

| **Parameter** | **Value** | | **Source** |
| --- | --- | --- | --- |
| Length | 200 | km | assumption |
| *Material intensity* |  |  |  |
| Aluminium conductor | 1400 | kg/km | Product specification |
| Insulation mass | 600 | kg/km | Product specification |

Table S2.2. Inventory for the high voltage cable.

| Parameter | Value |  | Source |
| --- | --- | --- | --- |
| Length | 20 | km | assumption |
| *Material intensity* |  |  |  |
| Aluminium conductor | 4136 | kg/km | Product specification |
| Insulation mass | 1772 | kg/km | Product specification |

Table S2.3. Inventory for the set of transformers.

| **Parameter** | **Value** | | **Source** |
| --- | --- | --- | --- |
| *Material intensity* |  |  |  |
| Copper | 18976 | kg | based on Vélez-Henao et al. (2021) |
| Steel | 29893 | kg | based on Vélez-Henao et al. (2021) |
| Transformer oil | 72653 | kg | based on Vélez-Henao et al. (2021) |

**Support Information 3**

FS3: Sensitivity Analysis of various parameters for wind turbine manufacturing. The results indicate the percentual changes of the overall baseline GHG emission result for the onshore wind farm.


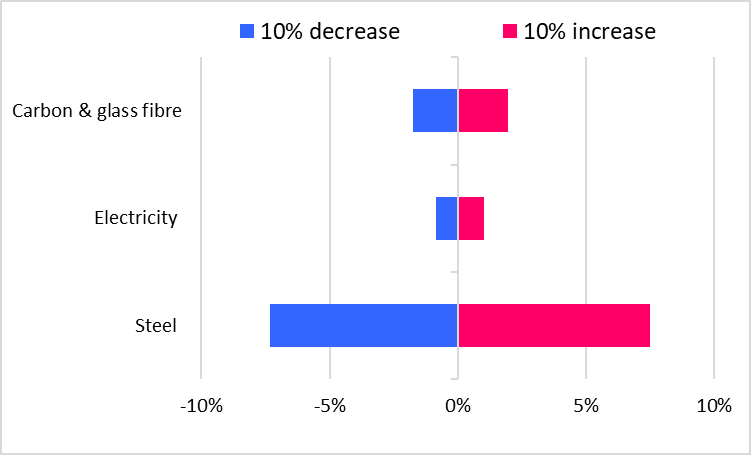

Supplement: Supplemental Material [file TNZR_A_2344785_SM0976.docx]
